# An investigation of the shelf life of cold brew coffee and the influence of extraction temperature using chemical, microbial, and sensory analysis

**DOI:** 10.1002/fsn3.3812

**Published:** 2024-01-24

**Authors:** Samuel N. Lopane, John U. McGregor, James R. Rieck

**Affiliations:** ^1^ Department of Food, Nutrition, and Packaging Sciences Clemson University Clemson South Carolina USA

**Keywords:** coffee, chemical analysis, cold brew, extraction, sensory

## Abstract

The shelf‐life of cold and hot water extraction coffees based on sensory and chemical profiles and microbial growth was examined, which also allowed the study of the influence of extraction temperature on the chemical and sensorial profiles of coffee. The shelf life of refrigerated cold‐ and hot‐brewed coffee was limited not by microbial stability but rather by deterioration in sensory attributes. Further work is recommended to elucidate the mechanisms of coffee staling in a refrigerated environment, with particular interest in the degradation products of chlorogenic acid, as a significant decline in chlorogenic acid concentration was found over the storage period. Cold‐extracted coffees were found to be chemically and sensorially different beverages from coffees extracted at high temperatures. Additionally, the cold‐brewed coffees had greater sensory flavor stability over the storage time than the hot‐brewed treatment.

**Practical application:**

This study advances the industry's understanding of the shelf life of ready‐to‐drink bottled cold coffees and demonstrates that lower brewing temperatures lead to greater flavor stability over shelf life. The findings also provide brewing parameters that can help guide product developers in modulating the flavor of commercial cold coffees.

## Graphical abstract

The shelf‐life of cold and hot water extraction coffees based on sensory and chemical profiles and microbial growth was examined, which also allowed the study of the influence of extraction temperature on the chemical and sensorial profiles of coffee. The shelf life of refrigerated cold‐ and hot‐brewed coffee was limited not by microbial stability but rather by deterioration in sensory attributes. Additionally, the cold‐brewed coffees had greater sensory flavor stability over the storage time than the hot‐brewed treatment.

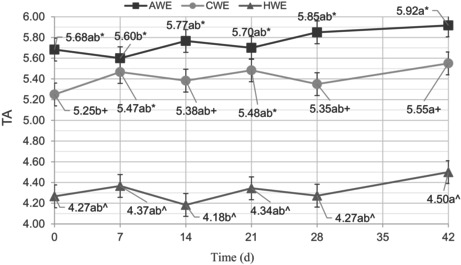

## 1 INTRODUCTION

### 1.1 Coffee overview

Coffee is a globally popular beverage, especially in the United States. According to a report from the National Coffee Association (NCA), in 2018, 64% of US consumers drink coffee daily (NCA, 2018). An increasing amount of this coffee consumption in the United States is specialty coffee, which can have varying definitions but is defined by the Specialty Coffee Association (SCA) as “coffee or coffee experience recognized for its distinctive attributes, and because of these attributes, has significant extra value in the marketplace” (Giuliano et al., 2021). This growth in specialty coffee consumption makes it clear that there is an increasing appreciation, interest, and demand from consumers for high‐quality coffees with unique flavors (Lynley, 2017). Coffee's global journey from seed to cup can be characterized by six main stages: growth, harvest, processing, roasting, grinding, and extraction. This study focused on the extraction of coffee, but it is important to emphasize that all the previous steps in the coffee journey govern the chemical and physical components of the coffee bean that will be extracted into the final beverage. Coffee extraction represents the selection (via solvation) of the chemical compounds that are created in the previous steps of coffee's journey to the consumer's cup.

### 1.2 Extraction details

Extracting coffee is the aqueous solvation of the flavorsome, soluble compounds in coffee, which are mainly aprotic charge‐neutral organic compounds, organic acids, and conjugate salts (Hendon et al., 2014). Extracting or brewing coffee is the attempt to dissolve as many pleasing flavors from the bean into the cup to create a balanced and tasty beverage, and this happens through the intimate contact of water with roasted coffee solids (Petracco, 2005).

Chemically, the polarity of water allows it to solvate the polar constituents of a coffee bean through either molecular or ionic dissolution (Bladyka, 2014; Wellinger et al., 2017). In addition to the solvation of soluble compounds, hydrolysis of larger and less soluble compounds also occurs and is dependent on the temperature and pressure of the extraction system (Cammenga et al., 1997). Nonpolar (hydrophobic) molecules will dissolve in water at low levels through the formation of hydrate cages (Hendon et al., 2014).

The soluble substances present in the roasted coffee seed vary widely, and not all compounds within coffee have desirable flavors. There are at least 1800 chemical constituents in coffee, many of which have different extraction rates and different flavors that they will contribute to the cup (Lee et al., 1992). Thus, extraction controls not just the concentration of the beverage but also the chemical composition. The percentage of coffee solids extracted is determined by the rate and quality of the extraction. The extraction rate is dependent on the specific extraction surface (particle size), the temperature of the solvent/solid interaction, the agitation of the brewing matrix, and the solid/solvent ratio (Rao, 2010; Uman et al., 2016). The final solute concentration is then determined by the extraction rate and the extraction time (Rao, 2010). The quality of the extraction is dependent on the slurry temperature, the distribution of the particle size, the contact size, the specific water chemistry, the extraction time, and the uniformity of the extraction. (Rao, 2010).

### 1.3 Cold brew extraction

Cold brew coffee is a product with growing market share—it can offer the potential for a unique flavor profile, a position within the growing specialty coffee world, and ready‐to‐drink convenience (Sisel, 2016). Products touted as cold brew coffee, also known in various places as “Dutch coffee”, are coffee beverages that are extracted using low temperatures and a longer time than traditional hot‐brewed drip coffee or espresso (Hwang et al., 2014). The different temperature of water gives cold water‐extracted (CWE) coffee an entirely different extraction profile, as different compounds are extracted at different rates and many coffee constituents have temperature‐dependent solubility (Bladyka, 2014). Many producers of CWE coffee claim that it has lower acidity, improved taste, and increased smoothness when compared to traditional hot‐brewed coffee (Sisel, 2016; Starbucks, 2018; Stumptown Coffee Roasters, 2018). However, there is little published data on the exact chemical characteristics of CWE coffee to verify these claims (Hwang et al., 2014). Cold brew coffee is most often produced either by dripping (percolation) or steeping (decoction) (Kim & Kim, 2014). Generally, these extractions can be carried out at different temperatures and still be considered CWE—some producers extract at ambient temperatures and some at refrigeration (Kim & Kim, 2014). These extractions can be carried out over different periods of time—often ranging anywhere from 3 to 24 h (Daeschel et al., 2017; Hwang et al., 2014). Another benefit of CWE coffee is that it can be canned or bottled in a ready‐to‐drink (RTD) format to be consumed later by the consumer (Sisel, 2016). This presents the potential problem of microbial and sensory shelf life.

### 1.4 Objectives

In this study, three extraction treatments of coffee were brewed, bottled, and analyzed over a period of 42 days. The shelf life of refrigerated cold‐ and hot‐brewed coffees was investigated based on sensory and chemical profiles and microbial growth, which also allowed the examination of the influence of extraction temperature on the chemical and sensory profiles. Because of the dual purpose of this study, the experiment was structured as a split‐plot design: multiple independent variables (time and extraction temperature) affected many dependent variables that were measured.

## 2 MATERIALS AND METHODS

### 2.1 Sample preparation

This study was divided into three treatment groups based on the extraction method of the same roasted and ground coffee. All three treatments used the same coffee, obtained from Due South Coffee (Greenville, SC, USA). All the coffee used for the three treatments was of the same geographic origin, processing method, roast level, and grind size. Specifically, it was Due South's Brazilian Black Diamond coffee, a natural‐processed coffee from the farm Vista Elegre in the Cerrado Mineiro region of Brazil, a region with altitudes varying between 800 and 1000 meters above sea level. The Arabica cultivar of the coffee used was Acaiá Cerrado, and it was imported to the US by Ally Coffee (Greenville, SC, USA). The tasting notes from the roaster were “Brown Sugar, Chocolate, and Smooth.” The coffee was roasted 6 days before extraction, and the roast degree was quantified with a Javalytics roast analyzer as Gourmet 72.9 (Madison Instrument Inc, Middleton, WI, USA). This coffee was chosen because it was actively being used to produce a cold brew available in the market. This study was conducted in Greenville, SC, USA.

The coffee used for all treatments was freshly ground using a Mahlkönig EK 43 (Mahlkönig, Hamburg, Germany) with a grinder burr aperture of 8, and the particle size was quantified using a standard set of coffee sieves. 50% of the ground particles were 710 μm, 35% were 850 μm, 5% were 600 μm, and another 5% were 1180 μm. To ensure a consistent extraction, all the water for extracting each treatment of coffee was standardized by adding the same ratio of minerals to distilled water using Third Wave Water mineralization packets (Third Wave Water, Cedarville, OH, USA). This resulted in water with a mineral concentration of 85 ppm [Mg^2+^], 45 ppm [Ca^2+^], and 20 ppm [HCO_3_^−^]. All treatments were prepared in triplicate and assigned a randomized three‐digit code, resulting in nine groups of samples.

#### 2.1.1 Cold water extraction

CWE coffee was prepared using an infusion method (full immersion). The coffee was ground to the particle size specified in Table 1 and filled into a Toddy cold brew commercial filter bag (Toddy LLC, Fort Collins, CO, USA). This bag was then placed in a 3‐gallon Cambro brewing vessel, and mineral‐standardized water (Table 2) at 4°C was added at a coffee‐to‐water ratio of 1:14. After an extraction time of 4 h in a refrigerator set to 4°C, the filter bag was removed, and the resulting coffee solution from each replicate was bottled.

**TABLE 1:** Schedule of analytical tests performed on coffee treatments.

Day	Analysis performed on all treatments
0	Refractive index/TDS, pH and titratable acidity, GC/MS, sensory analysis, and microbiological analysis
7	pH and titratable acidity, sensory analysis, and microbiological analysis
14	pH and titratable acidity, sensory analysis, and microbiological analysis
21	Refractive index/TDS, pH and titratable acidity, HPLC, GC/MS, and microbiological analysis
28	pH and titratable acidity, sensory analysis, and microbiological analysis
42	Refractive index/TDS, pH and titratable acidity, HPLC, GC/MS sensory analysis, and microbiological analysis

**TABLE 2:** Guide to sensory attributes (adapted from the WCR Sensory Lexicon) (World Coffee Research, 2016).

Attribute	Definition
Acidity	The fundamental taste factor associated with a citric acid solution
Sweetness	Fundamental taste sensation, of which sucrose is typical. Generally associated with sweet aroma descriptors such as fruity, chocolate, and caramel
Bitterness	The fundamental taste factor associated with a caffeine solution
Astringency	Characteristic of an after‐taste sensation consistent with a drying effect in the mouth. A drying, puckering, or tingling sensation on the surface and/or edge of the tongue and mouth
Longevity	Persistence of flavor in the mouth is the time that the full, integrated sensory experience sustains itself in the mouth and after swallowing
Papery‐Musty‐Stale‐Earthy	The flavor is characterized by a lack of freshness, the aromatic associated with white paper cups, and/or the somewhat sweet, heavy aromatic associated with decaying vegetation and damp, black soil
Sour‐Fermented Winey‐Over‐Ripe	The pungent, sweet, slightly sour, sometimes yeasty, alcohol‐like aromatic characteristic of fermented fruits or sugar or over‐proofed dough, and/or the sweet, slightly sour, damp, musty/earthy aromatic characteristic of fruit or vegetables past their optimum ripeness, and/or the sharp, pungent, somewhat fruity, alcohol‐like aromatic associated with wine
Chemical‐Rubber‐Skunky‐Medicinal	A clean, sterile aromatic characteristic of antiseptic‐like products such as band‐aids, alcohol, and iodine, and/or dark, heavy, slightly sharp, and pungent aromatics associated with rubber and/or a combination of aromatics associated with skunks
Vegetative‐Hay‐Herb Herb‐like	The lightly sweet, dry, dusty aromatic with a slight green character associated with dry grasses and/or the aromatic commonly associated with green herbs may be characterized as sweet, slightly pungent, and slightly bitter. May or may not include green or brown notes
Aroma Intensity	Strength of the aroma—evaluated by the smell as well as the intensity of the volatile flavor when drinking
Mouthfeel	The thick feeling of the beverage as you press your tongue through it

#### 2.1.2 Ambient water extraction

The ambient water extraction (AWE) coffee was prepared in the same way as the CWE, but at ambient (25°C) temperatures.

#### 2.1.3 Hot water extraction

Hot water extraction coffee was prepared by a hot drip method in a Bonavita BV1900TS (Bonavita, Seattle, WA, USA) at a coffee‐to‐water ratio of 1:18 and a water temperature of ~97°C. Immediately after brewing, the coffee was flash‐chilled by filling a retort bag and immersion into an ice bath (coffee chilled to 7°C in ~10 min). This method was chosen to match café methodology for preparing hot‐brewed coffee served cold after consulting with the supplier of coffee (Due South Coffee, Greenville, SC, USA).

#### 2.1.4 Bottling

All three treatments were performed in triplicate, and the samples from each group were bottled into fifty 120‐mL amber glass bottles, sealed with a plastic screw cap, labeled, and stored at 7°C. A randomization plan was created that determined which tests would be administered to each bottle at each date. An outline of the tests performed is in Table 1.

### 2.2 Analytical techniques

#### 2.2.1 Refractive index/total dissolved solids

The percentage of total dissolved solids (%TDS) in each of the samples was measured using a specialized coffee refractometer (Voice Systems Technology, Inc., Harvard, MA, USA) on 0, 7, 14, 21, 28, and 42 days. First, the refractometer was calibrated using distilled water, and then a clean plastic pipette was used to transfer 3 drops of the sample to the sample well of the refractometer. The sample was allowed to equilibrate, and then the %TDS was obtained and recorded.

#### 2.2.2 HPLC analysis

The concentration of a chlorogenic acid, 3‐O‐caffeoylquinic acid (5‐CQA, the latest IUPAC designation), in the brewed coffees in each of the sample groups was measured at days 21 and 42 using high‐performance liquid chromatography (HPLC) connected with a UV detector. Results were not able to be obtained on day 0 due to an equipment malfunction. Sample preparation was performed following the methods described in Gloess et al., 2013. Briefly, 2 g of the brewed coffee sample was added to 500 μL Carrez I (30% ZnSO_4_ aqueous solution), 500 μL Carrez II (15% potassium hexacyano ferrate trihydrate), 500 μL methanol, and then diluted with 25 mL H_2_O and filtered with a nylon Cole Parmer syringe filter (Cole Parmer, Vernon Hills, IL, USA). The samples were then analyzed in triplicate using a Waters 1525 binary HPLC pump with a Waters 717+ autosampler, degasser, and thermostat, and a Waters 2487 dual absorbance detector (Waters Corp, Milford, MA, USA). Mobile phase A was H_2_O with 0.1% formic acid, and mobile phase B was acetonitrile with 0.1% formic acid. The flow rate was 0.5 mL/minute in isocratic conditions at 30% for 40 min. The column used was a Gemini 5u C18 110A with a size of 150 mm × 4.60 mm × 5 μm (Phenomenex Inc, Torrance, CA, USA). For the analysis of 3‐O‐caffeoylquinic acid (5‐CQA), the detector was set to 325 nm. The compound was identified and quantified using commercially available HPLC‐grade standards that were <99% pure.

#### 2.2.3 pH and Titratable acidity

The pH and titratable acidity (TA) in the brewed coffees in each of the sample groups were measured at 0, 7, 14, 21, 28, and 42 days. The pH was measured in duplicate at 25°C using an Orion 420A pH meter, and calibrated using a two‐point calibration (Thermo Fisher Scientific, Waltham, MA, USA). For the titratable acidity, also measured in duplicate, 40 mL of each sample was titrated with 0.1 M NaOH to a pH of 8.0, and the volume of NaOH was recorded.

#### 2.2.4 Headspace analysis—GC/MS

The headspace intensity of each of the sample groups was measured at 0, 21, and 42 days using gas chromatography/mass spectrometry (GC/MS). Ten mL of each sample was placed into clean 15 mL GC–MS vials, capped with Teflon™‐lined septa, sealed, and then analyzed using a gas chromatograph/mass selective detector system (Hewlett Packard 7694 headspace sampler, Hewlett Packard HP 6890 series gas chromatograph, and Hewlett Packard HP 5973 mass selective detector (Hewlett Packard, Wilmington, DE, USA)). The sample was equilibrated in the autosampler for 30 min at 90°C, with a loop/valve temperature of 110°C and a transfer line temperature of 115°C. The gas headspace was then automatically injected onto a 30‐m‐long HP 5 MS capillary column with a 0.25 μm internal diameter. The GC oven was programmed for a time–temperature profile of: 35°C for 1 min, followed by a 3°C/min ramp to 100°C, then 5°C/min to 220°C, with a total runtime of 46.67 min. The data analysis was performed with HP Chem Chemstation Integrator, and the identification of volatiles was based on the comparison of retention times to authentic standards and computer matching of mass spectra to a reference library (Hewlett Packard). Although many compounds were identified, 11 compounds that have a majority contribution to typical coffee flavor, based on Flament and Bessière‐Thomas's work, were chosen and summed in order to study the headspace of the samples (Flament & Bessière‐Thomas, 2002). The compounds chosen were 2‐methyl Furan, 3‐methyl butanal, 2‐methyl butanal, 2,3 pentanedione, acetic acid, 2‐furan carboxaldehyde, 2,5 dimethyl pyrazine, 2‐furan carboxaldehyde, 5 methyl, 2 furan methanol, acetate, 2 ethyl‐6‐methyl pyrazine, and pyridine. The integrated intensities of the compounds chosen were then summed to give a headspace intensity (Gloess et al., 2013).

#### 2.2.5 Microbiological growth—APC and psychrotrophic count

All of the brewed coffees in each of the sample groups were tested for microbiological growth at 0, 7, 14, 21, 28, and 42 days. Enumeration of microbiological growth was achieved with a total aerobic plate count using standard plate count procedures as well as a psychrotrophic count. All plate counts were performed with 3 M Petrifilm and followed procedures as outlined in the 3 M Petrifilm Aerobic Count Plate Interpretation Guide and AOAC official method 990.12 (3 M, St. Paul, MN, USA). Aseptic technique was followed, and all inoculation and preparation of plates were performed under a laminar air flow hood. Briefly, 1 mL of each sample was placed onto the medium using a sterile pipette tip, and the inoculum was evenly distributed over a circular area using the 3 M Petrifilm spreader. The aerobic plate count was performed in duplicate, and two dilutions were used: 10^0^ and 10^−1^. For the aerobic plate count, the plates were then incubated for 48 h at 35°C, and any microbiological growth was enumerated on a colony counter following the counting guidelines in AOAC 990.12 (AOAC, 1994). For the psychrotrophic count, the plates were incubated for 10 days at 7°C and counted in the same manner.

#### 2.2.6 Sensory analysis

The following sensory study was reviewed and approved by the Clemson University IRB (Institutional Review Board). Administrators were trained and certified by Clemson University's IRB, and each subject provided informed consent prior to participation.

The sensorial quality shelf life of the coffee extracts was determined by the use of a blind sensory analysis, using a trained panel of five coffee sensory professionals to evaluate the sensory attributes of the coffees over a period of 42 days (adapted from methods in Gloess et al., 2013, Pérez‐Martínez et al., 2008, Stokes et al., 2017). The panel was made up of pre‐trained coffee sensory professionals from green coffee importer Ally Coffee and roaster Due South Coffee that are engaged in cupping coffee at least once a week. These employees are responsible for the purchasing decisions with regards to purchasing lots of coffee, and they rely on their sensory evaluation of brewed coffee to inform these decisions. Coffee cupping is a systematized sensory analysis protocol with a ballot created and maintained by the SCA (Specialty Coffee Association, Irvine, CA, USA). Using pre‐trained professionals reduced the amount of pre‐training that needed to be administered and allowed for a smaller panel to be used.

This sensory panel made extensive use of the World Coffee Research (WCR) Sensory Lexicon, “a universal language of coffee's sensory qualities, and tool for measuring them” (Chambers et al., 2016; World Coffee Research, 2016). This sensory lexicon “identifies 110 flavor, aroma, and texture attributes present in coffee, and provides references for measuring their intensity” (World Coffee Research, 2016, World Coffee Research, Portland, OR, USA). These references are all easily obtained, consistently manufactured products. Before the sensory analysis began, the authors conducted a training and calibration session that consisted of discussing the terms from the lexicon that would be used in this study, then tasting the commercially available references according to the coffee lexicon (for instance, the reference for a “sweet” score of 1.0 is a 1% sucrose solution). Then, the panelists tasted commercially available cold brews, and evaluated them according to the terms used in this study. The results were discussed as a group in order to align the panel and create a consensus. The panelists were well acquainted with the WCR Sensory Lexicon and were thus quickly able to align. This training was facilitated by a certified “Q” Instructor from the Coffee Quality Institute (Coffee Quality Institute, Aliso Viejo, CA).

The members of the panel evaluated all three treatment groups (extraction methods) in triplicate on each of the sampling dates (0, 7, 14, 28, and 42 days), totaling 9 samples evaluated by each panelist on each day. The evaluations all took place in Ally Coffee's cupping room. The samples were presented monadically, in a randomized order, in 120‐mL amber glass bottles labeled with 3‐digit randomly coded numbers and poured into a white porcelain cupping bowl. The serving temperature was 7°C. The panelists evaluated each sample for the intensity of eleven sensory attributes (Table 2) on a scale from 0 to 15 (0 = none, 2 = barely detectable, 6 = slightly intense, 8 = moderately intense, 10 = intense, 12–15 = very intense). Each of the judges' scores was compiled and statistically analyzed to create a sensory score for each sample. The sensory form that was used to evaluate the coffees can be found in Supplemental Info.

#### 2.2.7 Statistical analysis

A randomized design was used for the sampling and assigning of each of the 50 bottles of the three treatments in triplicate to each test on each sampling date. The mixed procedure from Statistical Analysis Software was used to analyze the data, and comparison of means was done using Fisher's least significant difference (LSD) method at a significance level of .05 (SAS Institute, Cary, NC USA). This experimental design, as well as the statistical techniques used, allowed the study of both the effect of time and the extraction method on the coffee beverages. For the purposes of this study, shelf life was examined by studying the effect of time—the changes occurring from day 0 to day 42—whereas the influence of extraction temperature and method was determined by examining the differences between the treatments.

## 3 RESULTS AND DISCUSSION

### 3.1 Effect of time—Significant microbial changes

In this study, there was no detectable bacterial growth in any of the samples. For all sampling dates (0, 7, 14, 21, 28, and 42 days), all samples were <25 CFU/mL for both the total aerobic count and the psychrotrophic count. This agrees with other established work on refrigerated coffee brews (Daeschel et al., 2017). This lack of microbial growth in coffee extracts stored at refrigeration temperatures is likely due to the multiple hurdles presented: scarcity of microbial nutrients, low pH (between 4.27 and 5.92), and other antimicrobial factors present within the coffee such as chlorogenic acids, caffeic acid, and caffeine (Almeida et al., 2006; Daeschel et al., 2017).

This study and Daeschel et al. (2017) only tested for vegetative growth, which is the primary concern for products that are held constantly at refrigeration but is not the only concern for products being sold unrefrigerated. Any cold brew being sold as a shelf‐stable canned product is classified as a low‐acid product; thus, it must be processed to prevent the growth of *C. botulinum*. Further work is recommended to study the effect of thermal processing on the sensory characteristics of bottled or canned unrefrigerated cold brew coffee.

### 3.2 Effect of time—Significant chemical changes

#### 3.2.1 pH and TA

**FIGURE 1:**
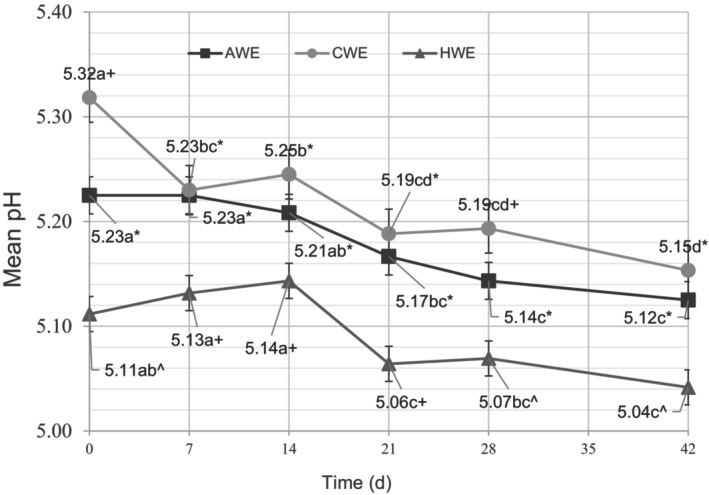
pH of Ambient Water Extraction (AWE), Cold Water Extraction (CWE), and Hot Water Extraction (HWE) treatment at days 0, 7, 14, 21, 28, and 42. Means not followed by the same letter are significantly different between sampling dates and means not followed by the same symbol are significantly different between treatments (*p* = .05), as determined by the Fisher's least significant difference (LSD) test.

**FIGURE 2:**
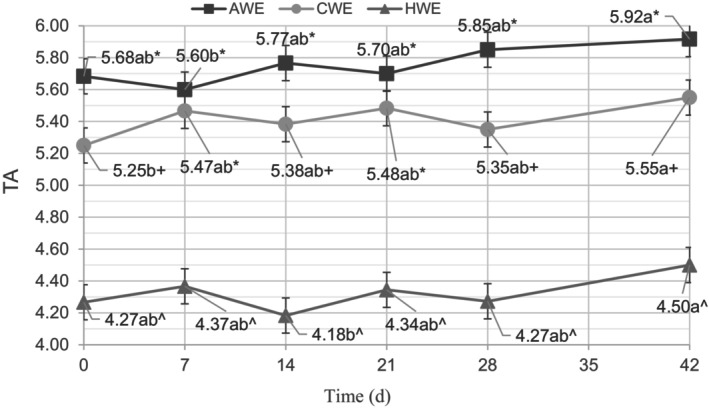
Titratable acidity (TA) of ambient water extraction (AWE), cold water extraction (CWE), and hot water extraction (HWE) at days 0, 7, 14, 21, 28, and 42, expressed in mL of 0.1 M NaOH added to 40 mL of sample. Means not followed by the same letter are significantly different between sampling dates and means not followed by the same symbol are significantly different between treatments (*p* = .05), as determined by the Fisher's least significant difference (LSD) test.

There was a statistically significant decrease in the mean pH of all three treatment groups from day 0 to day 42 (Figure 1). This indicates that over the 42‐day testing period, the concentration of H^+^ ions increased with time in all the coffee beverages. This agrees with previous work on the aging of coffee brews under refrigerated storage (Pérez‐Martínez et al., 2008); however, this study establishes this decrease in pH for coffee extracted at low temperatures. Dalla Rosa et al. (1990) proposed a certain pH (4.8) as a limit of acceptance for shelf‐life. However, more recent work from Pérez‐Martínez et al. (2008) on stored coffee brews suggested that this pH limit is not suitable to be applied to all coffees and all brewing methods, and “pH is not the only factor that determines the limit of acceptance of coffee brews” (Pérez‐Martínez et al., 2008). The results presented here agree with Perez‐Martinez's conclusions, as the coffees never reached the limit of acceptance proposed by Dalla Rosa et al. (1990), but they all had significantly increased off‐flavor sensory scores by the end of the testing period. Chemical analysis alone is not the best way to determine the shelf‐life of brewed coffees, and the results of this study validate the importance of sensory evaluation. Furthermore, when comparing pH values from this study to pH values from Pérez‐Martínez et al. (2008) work with Colombian Arabica coffee, there is considerable variance in pH values based on extraction method and coffee origin. The titratable acidity, which is a better measure of acid's contribution to the flavor profile of a beverage, also experienced an increase for all three treatments over the 42 days; however, the increase was only statistically significant for the CWE treatment (Figure 2).

#### 3.2.2 Chlorogenic acid

**FIGURE 3:**
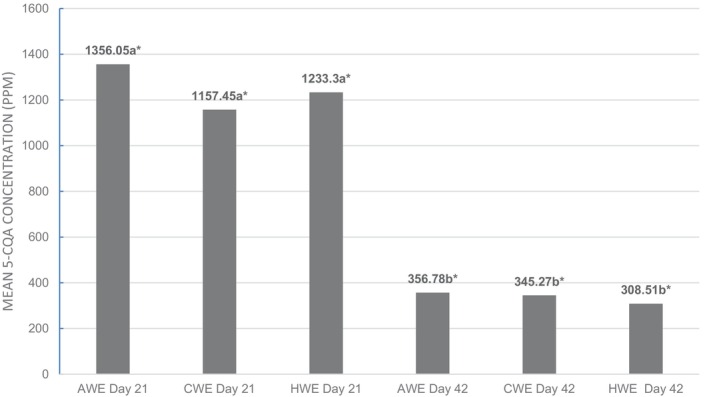
Concentration of 5‐CQA in ppm for each extraction treatment at days 21 and 42, expressed in ppm. Means not followed by the same letter are significantly different between sampling dates and means not followed by the same symbol are significantly different between treatments (*p* = .05), as determined by the Fisher's least significant difference (LSD) test.

The mean concentration of 3‐O‐caffeoylquinic acid (5‐CQA) in CWE, AWE, and HWE was significantly different between days 21 and 42, demonstrating a significant decrease over time in each treatment group, showing that time of storage had an effect on chlorogenic acid concentration in the coffee beverages (Figure 3). The thermal degradation of chlorogenic acid into caffeic and quinic acids is known to cause the development of sourness (Clarke & Vitzthum, 2008). This effect has mainly been demonstrated at elevated temperatures, and some have suggested that this would not occur at refrigeration temperatures (Pérez‐Martínez et al., 2008). The effect does increase with time, and the length of storage may make up for the lack of elevated storage temperatures in this study. One possibility for further investigation is the possibility that the decrease in 5‐CQA observed in this study was due to a low‐temperature hydrolysis of CQA into caffeic and quinic acids, potentially contributing to the staling and loss of quality in the beverages over time. An increase in quinic acid in stale coffee has been indicated to be one of the compounds responsible for increasing sour perception in stale brewed coffee held at high temperatures (Van der Stegen & Van Duijn, 1987). However, without analyzing the degradation products of 5‐CQA, it is not known for certain if this decrease in concentration of chlorogenic acid was the cause of the increase in off‐flavor scores.

#### 3.2.3 Headspace intensity

For the mean headspace intensity, measured in intensity counts, each treatment group did not exhibit a significant change across the three sampling dates—demonstrating that the shelf life did not influence the overall headspace intensity of the brewed coffees (Figure 7).

#### 3.2.4 Effect of time—Significant sensory changes

**FIGURE 4:**
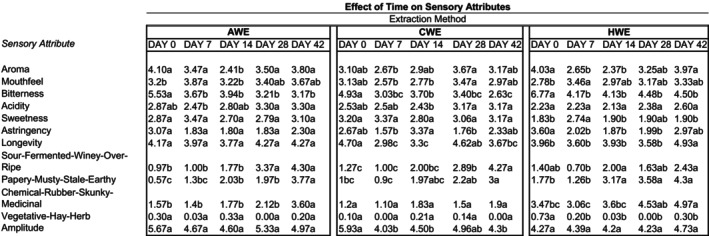
Mean values for sensory attributes of all three treatments over 42 days, showing the effect of time on sensory attributes. Means within the same row and treatment grouping not followed by the same letter are significantly different (*p* = .05), as determined by the Fisher's least significant difference (LSD) test.

**FIGURE 5:**
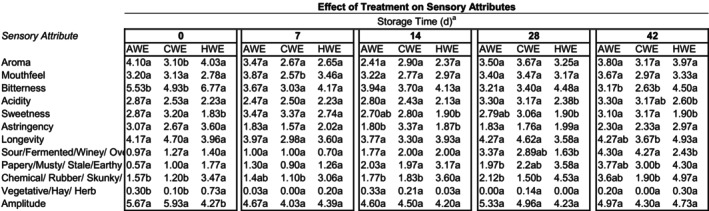
Mean values for sensory attributes of all three treatments on the five sampling dates, showing the effect of treatment on sensory attributes. ^a^Means within the same row and day grouping not followed by the same letter are significantly different. (*p* = .05), as determined by the Fisher's least significant difference (LSD) test.

The results of the sensory analysis of the coffees are summarized in Figures 4 and 5. The storage time had significant effects on the flavor profile for all treatment groups. For CWE, AWE, and HWE, the bitterness scores were significantly lower on day 0 than on day 42. Sweetness, astringency, and longevity mean scores fluctuated but did not have any clear trends over time. The most significant changes over the storage time that were consistent across treatments were the decrease in mean bitterness score and the increase in mean score for sour‐fermented‐winey‐overripe, and papery‐musty‐stale‐earthy. There was a clear relationship between storage time and the decreasing bitterness score, as well as a sour and papery defect score for all treatments. It is quite possible that the decrease in mean bitterness score was caused by the interaction of bitterness and sour perception—meaning the increase in sour off‐flavor dulled the perception of bitterness. There were other significant changes from storage time, but these are the changes that were common to all three treatments.

This increase in off‐flavors over time in the samples was clear and lines up with previous work on stored coffee brews (Dalla Rosa et al., 1990; Pérez‐Martínez et al., 2008). Storage effects are well documented in hot‐brewed coffees, and these results confirm that the sensory deterioration of cold brew with storage time is similar, but not identical, to hot‐brewed coffees. It is unclear what the exact chemical cause of these sensory changes is, but these results are helpful, as they show the development of these defects over time, even at refrigerated storage.

The cold brew treatments did not deteriorate in the same manner or time as the hot‐brewed treatments. This is demonstrated by the effect of the extraction method on defect scores: there were some notable differences in the staling of the HWE vs. AWE and CWE. Notably, HWE had significantly higher chemical scores overall, and sour scores were significantly higher for the cold brew extractions at the last sampling date (day 42), perhaps suggesting that the chemical‐rubber‐skunky‐medicinal flavor is more likely to develop in hot‐brewed coffees, whereas sour‐fermented‐winey‐overripe is more likely to develop in colder brewed coffees (Figure 4). This suggests that coffees brewed at low temperatures may have a longer organoleptic shelf life than coffees that have been brewed at high temperatures and then cooled. This may be due to the multitude of staling reactions that increase with rising extraction temperatures (Clarke & Vitzthum, 2008).

### 3.3 Effect of extraction temperature and method—Chemical profile

The other purpose of this study was to investigate the influence of extraction temperature on the chemical and sensory profiles of CWE coffee. Chemically, there were several differences between the hot‐brewed coffee and the two cold‐brewed coffees.

#### 3.3.1 %TDS

**FIGURE 6:**
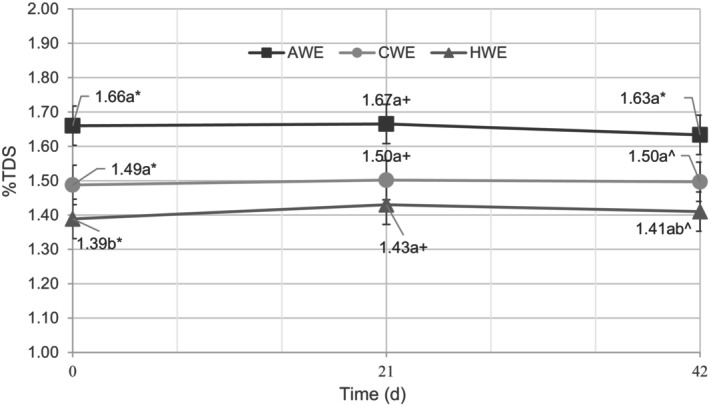
Graph of the concentration of total dissolved solids in each extraction treatment at days 0, 21, and 42, expressed in %TDS. Means not followed by the same letter are significantly different between sampling dates and means not followed by the same symbol are significantly different between treatments (*p* = .05), as determined by the Fisher's least significant difference (LSD) test.

One of the differences between the variables was the strength of the beverages. The AWE had the highest %TDS followed by the CWE and then the HWE (Figure 6). %TDS is the measure of how many solids are dissolved in the aqueous solution; thus, many other chemical properties are highly correlated with it. When interpreting the results of this study, it is important to keep in mind the impact that TDS has on all the properties that were measured. For instance, Frost et al. (2020) demonstrated that intensity measured via sensory evaluation and TDS were inversely correlated, whereas bitterness was positively correlated. All groups used the same grind size, but the HWE treatment group used a lower water‐to‐coffee ratio than the two immersion methods, so the difference in %TDS between the hot brew and the cold brew cannot be entirely attributed to the influence of extraction temperature. However, all the brewing parameters were identical between AWE and CWE in order to isolate the effect of the temperature of the brewing. The higher concentration of soluble solids in the AWE coffees, brewed at 25°C, than the CWE coffees, brewed at 4°C, agreed with previous literature that higher temperatures increase the extraction rate (Rao, 2010; Uman et al., 2016).

#### 3.3.2 pH and TA

The extraction temperature also significantly influenced the pH and TA of the coffee extracts (Figures 1 and 2). The HWE had a significantly lower pH than the two cold‐brewed coffee extracts. The CWE had the highest pH, with the AWE in the middle, so for these extractions, the higher the temperature of the extraction, the lower the pH of the resulting extract. The titratable acidity reversed the trend of pH, though, as the HWE had the lowest titratable acidity, followed by the CWE and then the AWE. This means that although the HWE had a greater free hydrogen ion concentration, it had a lower total acidity, as measured by TA, than the two cold‐brewed extracts. Titratable acidity is “a better predictor of acid's impact on the on flavor than pH,” so it follows that the two cold‐brewed treatments with higher TA ended the study with a significantly higher mean sour sensory score than the HWE group (Nielsen, 2003). This reversal of pH and TA results is worth considering further—the cold brew treatments had higher TA but a lower hydrogen ion concentration, and inversely, the hot brew treatments had lower TA and a higher hydrogen ion concentration. From these two results, it seems that the higher brewing temperature of the HWE extracted more completely dissociated acids (leading to a low pH and low acidic flavor impact), whereas the cold brew extracted more weak acids, which have lower levels of completely dissociated ions, leading to a higher pH and high TA and a higher flavor impact.

The low H^+^ concentration and high TA of the AWE and CWE could also be a function of the concentration of total dissolved solids in each of the coffee extracts. The TDS decreased from AWE to CWE to HWE, and similarly, the TA decreased from AWE to CWE to HWE. The higher TA in CWE and AWE could have simply been because those two solutions had more compounds solvated per mL of water. Results from the Gloess et al. (2013) comparison of extraction methods support this: “In this study [based on one single type of coffee], a positive correlation of the refractive index (°Brix) with the following attributes was observed: concentration of total solids, headspace intensity, concentration of caffeine, 3‐CQA and 5‐CQA, and titratable acidity.” When comparing extraction methods, the total concentration of a coffee solution will dictate many of the other chemical parameters.

#### 3.3.3 Headspace intensity

**FIGURE 7:**
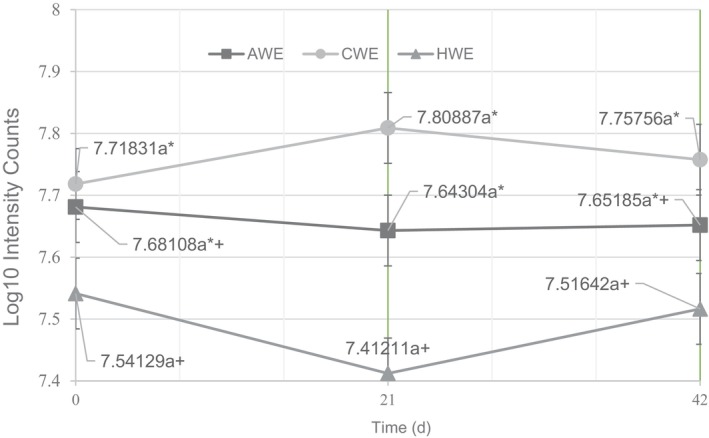
Graph of the headspace intensity of each extraction treatment at days 0, 21, and 42, expressed in log_10_ intensity counts.

The extraction temperature also influenced the headspace intensity of the coffee extracts. The CWE and AWE had higher mean headspace intensity on all three sampling dates than the HWE, and for the CWE, this difference was significant (Figure 7). Much of the appreciated flavor of coffee is present in the aroma/headspace of the brew, so it is interesting to consider that the lower temperature and longer time extractions had a higher concentration of volatiles. Further work needs to be done to elucidate these differences, as HS intensity is a rough measure of volatile concentration and is heavily influenced by the strength (TDS) of the solution and the selection of the compounds for sampling. Additionally, contrary to the findings of Hwang et al. (2014), acetic acid was detected in the headspace of all three extraction methods, demonstrating that a lack of acetic acid is not a defining feature of coffee brewed at low temperatures.

### 3.4 Effect of extraction temperature and method—Sensory profile

The extraction method of the coffee did significantly affect multiple sensory attributes of the coffee beverages, resulting in three products with distinct organoleptic characteristics. CWE and AWE had significantly higher mean sweetness scores and significantly lower mean bitterness scores than the traditionally extracted HWE at days 0 and 42. These sensory data suggest that cold brewing does create a beverage distinct from traditional hot‐brewed coffee that is consumed immediately or after cold storage for extended periods of time.

Another attribute of the coffee extracts that the extraction method influenced were the off‐flavors at the end of the 42‐day period (Figure 5). Based on the higher chemical scores for the HWE treatment on day 42, and the significantly higher sour scores for CWE and AWE on day 42 the data suggest that the chemical‐rubber‐skunky‐medicinal defect was more likely to develop over the storage time in hot‐brewed coffees, whereas the sour‐fermented‐winey‐overripe is more likely to develop over the storage time in colder brewed coffees. Further investigation is needed, but the sensory results also may point to low‐temperature extraction having greater flavor stability, as the colder extraction reduced the flavor deterioration of the brewed coffee. For example, at day 42, for both chemical and papery defects, the CWE mean is significantly lower than the HWE, but the AWE mean is not significantly different from either. In many cases, the means for sensory attributes between CWE and AWE could not be separated, meaning the flavor profile between the different methods of making cold brew was very similar. The largest difference between the two temperatures of extraction that are both considered cold brew may be the difference in deterioration. A limitation of the application of these results is the use of a natural processed coffee, which does differ from a washed coffee in its production and drying method, and thus the brewed product may experience different chemical and sensory changes over time and between extraction temperatures. Additionally, the HWE treatment did utilize the drip method, requiring the use of a different brew ratio, which does limit the comparison between the hot and cold water extraction treatments. Further work is recommended to isolate the variable of temperature in studying bottled cold brews.

## 4 CONCLUSION

Cold‐extracted coffees (CWE and AWE) were found to be chemically and organoleptically different beverages from hot‐brewed coffees. For flavor profile, the cold‐extracted coffees had significantly higher sweetness and lower bitterness than the hot‐extracted coffee on days 0 and 42. The cold‐extracted coffees also had greater flavor stability, as they exhibited a more gradual increase in off‐flavor scores and significantly lower defect scores for sour‐fermented‐winey‐overripe on day 42. Regarding the effect of time on bottled coffees, based on the findings in this study, the refrigerated shelf life of hot‐ and cold‐brewed coffee is limited not by microbial stability but rather by deterioration in sensory attributes, reinforcing the importance of sensory evaluation in quality control. Further work is recommended to elucidate the mechanisms of coffee staling in a refrigerated environment, with particular interest in the degradation products of chlorogenic acid, as a significant decline in 5‐CQA concentration was found over the storage period.

## AUTHOR CONTRIBUTIONS

**Samuel N Lopane:** Conceptualization (equal); data curation (supporting); formal analysis (supporting); investigation (equal); methodology (equal); supervision (equal); writing – original draft (equal); writing – review and editing (equal). **John U McGregor:** Conceptualization (equal); funding acquisition (lead); methodology (equal); project administration (equal); supervision (equal). **James R Rieck:** Data curation (lead); formal analysis (equal); software (lead).

## FUNDING INFORMATION

Funding was provided by the Clemson University Department of Food, Nutrition, and Packaging Sciences ‐ 55 Exchange Student Enterprise.

## CONFLICT OF INTEREST STATEMENT

The authors have no conflicts of interest to declare.

## ETHICS STATEMENT

The following sensory study was reviewed and approved by the Clemson University IRB (Institutional Review Board). All panelists participated voluntarily, and panelist consent forms were signed before the study began.

## Supporting information


Data S1.

## Data Availability

The data that support the findings of this study are available on request from the corresponding author. The data are not publicly available due to privacy restrictions.
