# Supplementary material for: An investigation of the shelf life of cold brew coffee and the influence of extraction temperature using chemical, microbial, and sensory analysis
Source: Food Sci Nutr. 2024 Jan 24;12(2):985–96. doi: 10.1002/fsn3.3812 (PMC10867521; doi:10.1002/fsn3.3812)
Supplement: Supplementary file 1 — Data S1. [file FSN3-12-985-s001.docx]

**Supplemental Information**

**Figure 1.** Ballot used by sensory panelists to evaluate coffee samples.

**-------------------------------------------------------------------------------------------------------------**

**Sensory Evaluation Form – Cold Brew Coffee**

**DATE _______________________**

**SAMPLE CODE _______________**

**PANELIST NUMBER___________**

Please evaluate each coffee sample on the intensity of the twelve sensory attributes, using a scale from 0 to 15. Please circle the line that represents the score you would like to give the intensity of the attribute. Between samples, please cleanse your palate.

**AROMA**

Overall Aroma Intensity

**
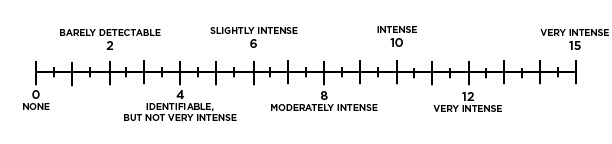
**

**TEXTURE**

Mouthfeel-Thickness

**
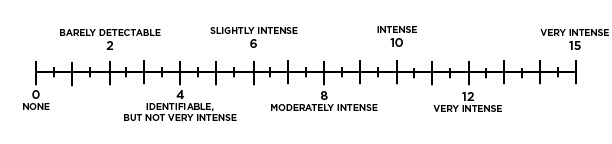
**

**TASTE / FLAVOR SPECTRUM**

Bitterness

**
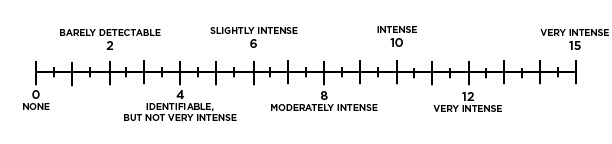
**

Acidity

**
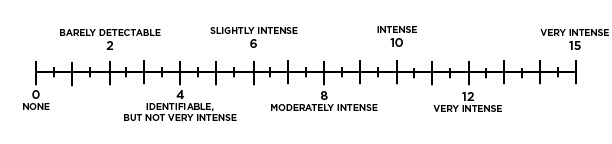
**

Sweetness

**
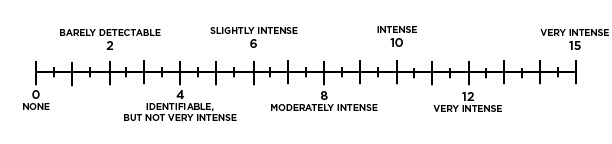
**

Astringency

**
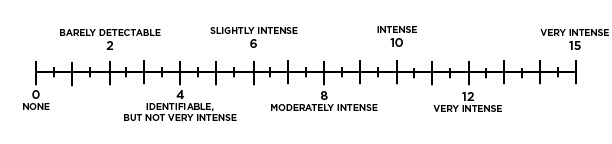
**

Longevity

**
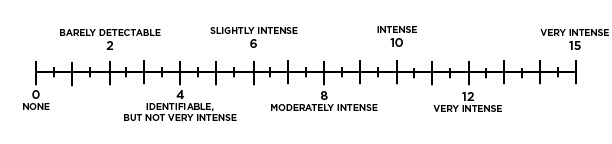
**

**Off-Flavors**

Sour-Fermented-Winey-Over-Ripe

**
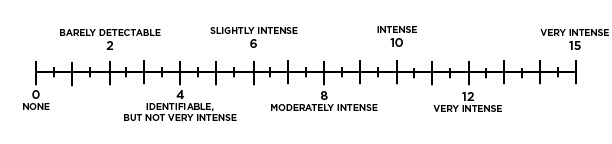
**

Papery-Musty-Stale-Earthy

**
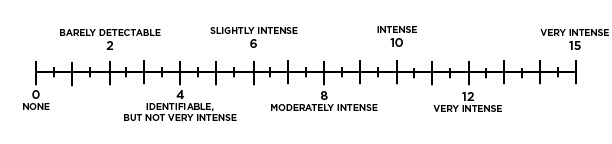
**

Chemical-Rubber-Skunky-Medicinal

**
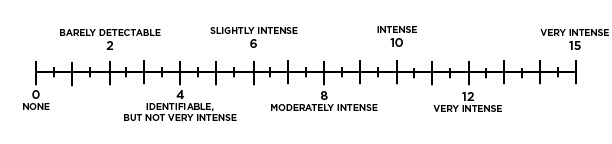
**

Vegetative-Hay-Herb

**
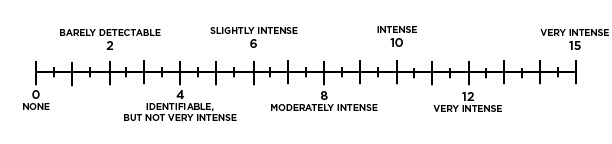
**
